# Hybrid synthesis of zirconium oxycarbide nanopowders with defined and controlled composition

**DOI:** 10.1039/c8ra09584a

**Published:** 2019-01-23

**Authors:** Daniel Hauser, Andrea Auer, Julia Kunze-Liebhäuser, Sabine Schwarz, Johannes Bernardi, Simon Penner

**Affiliations:** a Institute of Physical Chemistry, University of Innsbruck, Innrain 52c, A-6020 Innsbruck, Austria. Email: simon.penner@uibk.ac.at; Tel: +43 512 507 58003; b University Service Centre for Transmission Electron Microscopy, Technische Universität Wien, Wiedner Hauptstrasse 4-6, A-1040 Wien, Austria

## Abstract

A combined synthesis strategy involving a carbothermal reduction and gelation approach with glycine as gelating agent was used to obtain Zr-based (oxy)carbide materials with defined and controlled composition. A comparatively low temperature approach (1500 °C) allows exploration of the ZrC–ZrO_2_ phase diagram and reproducibly leads to zirconium (oxy)carbide phases with different C/Zr ratios, as confirmed by combined X-ray diffraction (XRD) and transmission electron microscopy (TEM) data. The latter also indicates a chemically very homogeneous distribution of oxygen and carbon throughout the sample bulk, a prerequisite for further characterization of its intrinsic physico-chemical properties. Due to the general variability of the synthesis procedure – variation of metal precursor, amount of gelating agent and carbon precursor source – it is expected that the method can be easily adapted and transferred to other metal – oxycarbide materials.

## Abstract 2

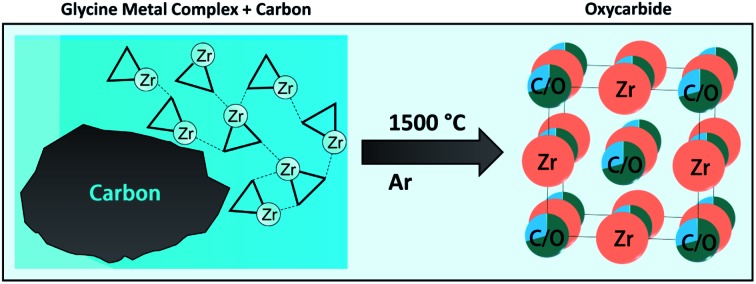
A combined synthesis strategy involving a carbothermal reduction and gelation approach with glycine as gelating agent was used to obtain Zr-based (oxy)carbide materials with defined and controlled composition.

## 1. Introduction

Metal carbides are used in a variety of different applications, especially for ultra-high temperature and high strength applications. The term “carbide” defines a material, which is composed of carbon and a less electronegative element. Often metal carbides, which will be usually labeled as “M_*x*_C_*y*_”, where *x* and *y* indicate a variable composition, are used.^1^ Transition metal carbides like TiC, Mo_2_C or ZrC present some auspicious properties like good elasticity, hardness, chemical resistance, thermal conductivity and optical properties like infrared reflection and absorption of visible light. Promising catalytic and electro-catalytic activities of ZrC and other transition metal carbides for C1 conversion have been investigated recently.^2^ A feature which is also crucial for catalytic applications, is that carbides, and here especially zirconium carbide as a representative material, tend to show a great affinity to oxygen, which is difficult to control. Consequently, it is also not straightforward to produce carbides in a pure oxygen-free composition. Residue oxygen in the carbide lattice can lead to so called oxycarbide materials, which exhibit the same cubic unit cell (*Fm*3[combining macron]*m*) and accordingly similar lattice parameters.^3^ To emphasize the importance of oxycarbide materials in catalytic research, it has been previously reported that for gasoline-operated fuel cells, a Mo-based oxycarbide is in fact the catalytically active phase.^4^ Generally, this material class has already been shown to have potential for applications as electrode material for various kinds of fuel cells, including intermediate temperature solid oxide and direct ethanol fuel cells.^5^

Several of these mixed transition metal-based oxidic/carbidic compounds are known, including the archetypical materials “TiC_*x*_O_*y*_” and “MoC_*x*_O_*y*_”, *x* and *y* thereby denoting the variable composition. Little is generally known about the oxycarbide phases of the transition metal zirconium.^6^ Those zirconium oxycarbides represent a solid solution of ZrC and ZrO_2_ and beneficially combine the properties of both materials – carbides in general have a relatively good electrical conductivity,^7^,^8^ while ZrO_2_ contributes a high ceramic stability and is at the same time a commonly used catalyst and support material in catalysis. As for other (oxy)carbide materials, application *e.g.* as electrode material in SOFC's could be envisioned due to its potentially beneficial conduction properties.

The standard commercial method for producing zirconium (oxy)carbides is a solid-state synthesis routine which needs high temperatures (around 2000 °C) and pressures to achieve the necessary high solid state diffusion rates.^9^ However, due to the need for these high temperatures, degradation of the specific area of the prepared powder will inevitably occur.^10^ In the carbothermal reaction, monitoring the carbon amount is crucial to control the composition for synthesis. The reaction mechanism has been thoroughly investigated and described in detail in ref. 9, 11 and 12.

Other methods like spark plasma sintering or solid-state synthesis with carbide precursors are known but are equally demanding in terms of synthesis conditions. Electrochemical and mechanochemical methods also offer a convenient and rather easy access to carbide materials.^13^–^16^ As a completely different method, the gelation procedure is long in use as a rather easy approach to synthesize a plethora of different ceramics, including also carbides.^17^ Such synthesis strategies for zirconium carbide and oxycarbides often rely on the use of organic solvents and reactants, which are more complex and environmentally less friendly in comparison to a water-based synthesis.^18^ One challenge, however, is the formation of oxo-zirconium species in aqueous solution.^19^ In an acidic solution with glycine ligands, nevertheless, the species are stabilized by the formation of zirconium–glycine clusters, before they further transform into a polymeric gel at the end of the gelation process.^20^

In this work, oxo-zirconium species are evenly distributed in a water-based solution and ligated, and therefore immobilized, by an organic network. This network is built by the amino acid glycine, which serves both as fuel for the auto-combustion and as ligand. An atomic level mixing of the reagents and flame temperatures of over 2000 °C result in an extremely fast and complete reaction. With this method, zirconium carbides and oxycarbides are synthesized by a gelation auto-combustion under inert atmosphere (Ar). The challenge hereby lies in the fine tuning of the stoichiometric ratios between oxometal ions, carbon content and ligands. Since the glycine content is affecting the gelation (gel agent), the combustion (fuel) and the composition (carbon source) at the same time, it cannot be controlled in a sufficient manner to fulfill all three requirements simultaneously. Therefore, the idea was obvious to add an additional carbon source with a high specific surface (*i.e.* activated carbon), which is borrowed from the carburization of ZrO_2_ to ZrC. The integration of the active carbon in the gel network does not affect the combustion and is essential for achieving phase-pure products with defined composition. Approaches without additional carbon always show additional oxide phases.

We show here that the described synthesis routine allows for reproducible and straightforward preparation of zirconium oxycarbide materials not only with defined composition and variable C/Zr ratios, but especially also at lower C/Zr ratios. These are notoriously difficult to stabilize as a phase-pure material due the formation of parasitic oxide phases. A combination of X-ray diffraction (XRD) and analytical electron microscopy (TEM) is used to monitor and judge the success of the synthesis routine.

## 2. Experimental details

### 2.1. Synthesis procedure

The synthesis strategy involves a combination of a carbothermal reduction route and a gelation approach, which was used to obtain a phase pure product at relatively low temperatures (1500 °C). This is based on a liquid–solid synthesis procedure, which involves the use of high surface carbon. As starting materials, zirconyl chloride octahydrate (ZrOCl_2_·8H_2_O, purity 99% Fluka Chemika), glycine (C_2_H_5_NO_2_, purity >99% Sigma Aldrich) and amorphous activated carbon (YP-50F, 1600 m^2^ g^–1^ Kuraray) were used. Gases were supplied by Messer (Ar 5.0).

**Fig. 1:**
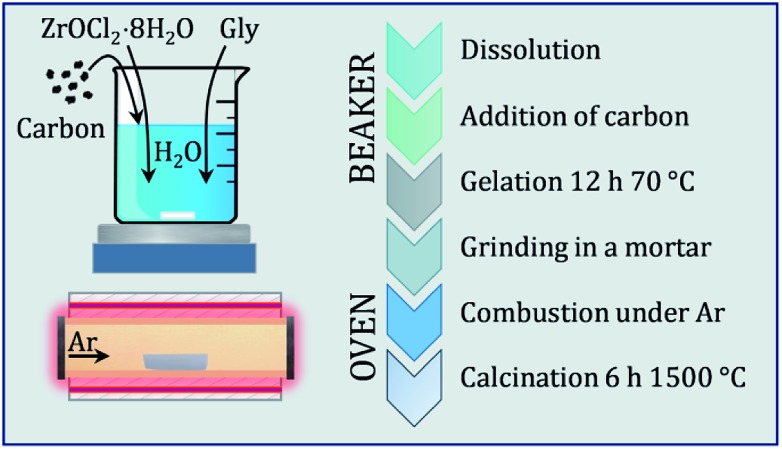
Flowchart diagram of the synthesis procedure, which is sectioned into a beaker workflow and the thermal treatment in the furnace.

A solution was prepared by dissolving zirconyl chloride octahydrate in a minimum amount of boiling water, according to its solubility product (approx. 30 mL g^–1^). As chelating agent and fuel, glycine was added to this solution, which is then stirred until solvation. As oxidizer, nitrate was added in form of concentrated nitric acid (68%), according to a molar ratio of metal : nitrate : glycine of 1 : 4 : 4. Glycine is known for thermal decomposition above 210 °C into different gaseous reactants.^21^ In a glycine–nitrate combustion, N_2_, CO_2_ and H_2_O are evolving as gaseous products, while carbon residues (up to 1.5 molar equivalents depending on the nitrate valence) can remain. Activated carbon was added to the clear solution in a molar ratio range to zirconium of 2 to 6 to minimize diffusion distances and enhance the conversion. While being stirred, the black suspension is converted to a gel by heating at 70 °C for 6 h. The dry gel could in principle be ignited at 250 °C and would burn completely off through auto-combustion. This would lead to a mixture of carbide, oxide and oxycarbide phases. Instead, the gel was ground to a finely dispersed homogeneous powder, which was transferred to an alumina boat and calcinated in a tube furnace at 1500 °C (5 °C min^–1^ rate) for 6 h under flowing Ar (200 sccm at 1.5 bar), so the auto combustion could occur under a non-oxidizing atmosphere. The impregnation of carbon with the oxo-zirconium–glycine network leads to a better conversion of ZrO_2_ to ZrC and zirconium (oxy)carbide, although with excess residual carbon. Full conversion was reached with molar C : Zr ratios of 2.5 and 6. The black powders are stored in closed glass vials under inert gas prior to analysis. The synthesis routine is schematically depicted in a comprehensive flowchart in Fig. 1.

### 2.2. Powder characterization

X-ray diffraction (XRD) data are recorded using a Stadi P Transmission-XRD, equipped with a Mythen1K detector and Mo K_α1_ X-ray source (*λ* = 0.7093 Å, 50 kV, 40 mA) and operated in the moving PSD mode with a step size of 6° (100 s per step). Lattice parameters and density of the powder samples were obtained by Rietveld refinement data of long-time measurements using the program TOPAS. BET data are collected with a Quantachrome Nova 2000 Surface Area and Pore Size Analyzer in a standard procedure by adsorption of liquid nitrogen.

A Thermo Scientific MultiLab 2000 spectrometer was used for X-ray photoelectron spectroscopy (XPS) characterization of the powder surface. Equipped with an ion-getter pump, the base pressure of the chamber is in the 10^–11^ mbar range. A monochromated Al Kα X-ray source (1486.6 eV) and an Alpha 110 hemispherical sector analyzer with seven channel electron multipliers were employed for the analysis. Charge compensation was performed by a flood gun, which provides electrons (6 eV) to the sample. The sp^2^-carbon component from adventitious carbon was used for energy shift correction for all spectra. For background correction, a Shirley-type was used, while peaks are fitted with Gaussian–Lorentzian line shapes with a 30% Lorentzian contribution. For the asymmetric carbide peak form, which is caused by shake-up like events as a result of the electrical conductivity of zirconium carbide,^22^ a tailing factor of 0.9 was applied to the GL(30) shape.

The lacey carbon film-supported powder samples were investigated with a 200 kV FEI Tecnai F20 S-TWIN high resolution analytical transmission electron microscope. Equipped with an EDAX Apollo XLT2 silicon-drift detector, energy-dispersive X-ray spectra (EDX) were collected.

## 3. Results and discussion

### 3.1. Structural characterization

**Fig. 2:**
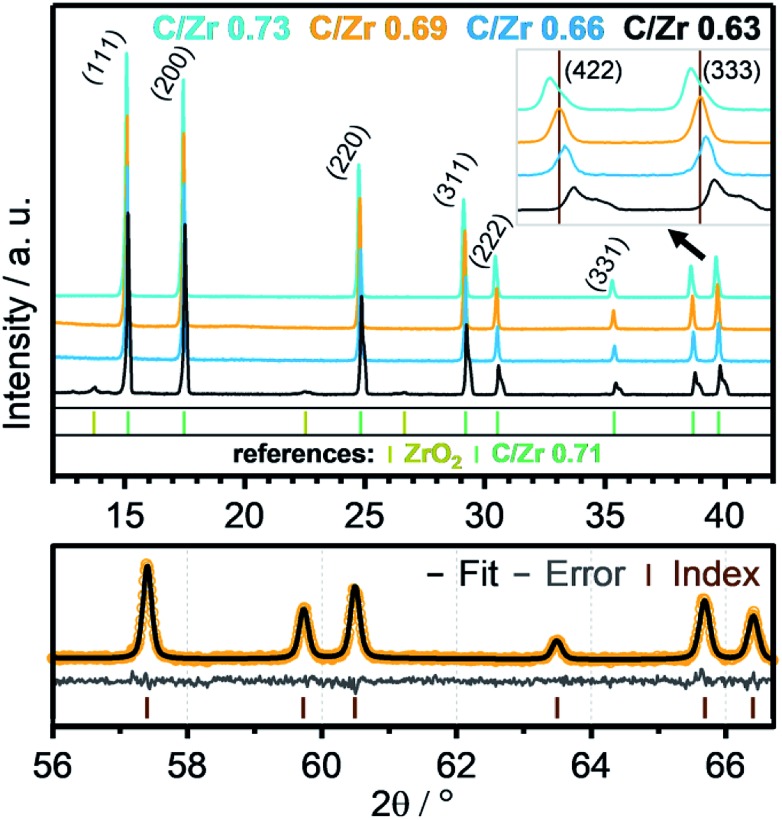
XRD data of four zirconium oxycarbide powder samples with different carbon content (C/Zr ratios = 0.73 (cyan), 0.69 (orange), 0.66 (blue), 0.63 (black)) after heat treatment at 1500 °C for 12 hours. An enlargement of the (422) and (333) reflexes, which are emerging at around 2*θ* = 39°, is shown in the right upper corner. Rietveld refinement for the high 2*θ*-values (56–66.5) of the phase with a C/Zr ratio of 0.69 is shown in the bottom part. The index bars indicate the peaks of the refined structure, as derived from Rietveld analysis.

**Fig. 3:**
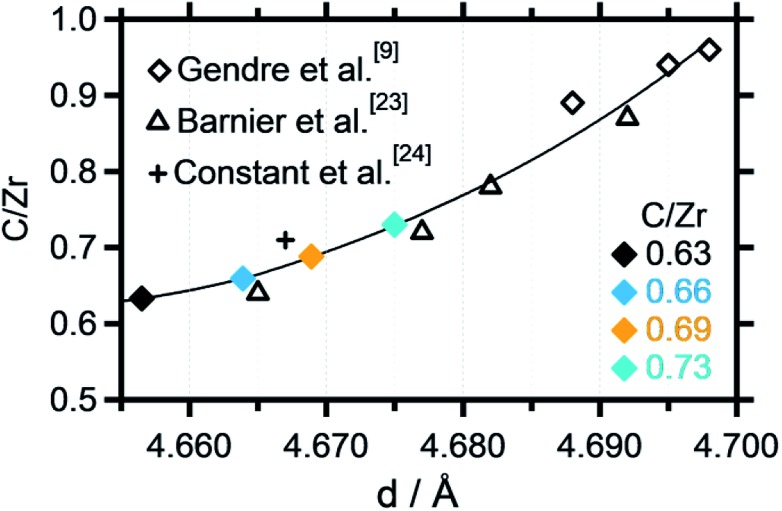
Zr-normalized carbon content plotted *vs.* the cell parameter extracted from the Rietveld refinement. A polynomial fit has been applied to fit the data points, which were partly taken from literature data.^9^,^23^,^24^

Recent literature reports indicate stability of zirconium oxycarbides in the ratio range between C/Zr = 0.64 and C/Zr = 0.98.^23^ On this basis, different precursor solutions were synthesized with controlled composition and phase purity. The XRD data of four representative product powders with different carbon amounts (corresponding to C/Zr ratios of 0.73, 0.69, 0.66, 0.63 – exact determination see below) are shown in Fig. 2. An introductory note on the determination of the composition of the oxycarbides should be given at this stage. In order to determine the C/Zr ratio of the differently synthesized materials, we followed a two-stage approach. The variation in the C/Zr ratio *via* synthesis has been achieved by using different molar amounts of activated carbon (and zirconyl chloride). In a second step, XRD data were collected for all samples and the diffractograms subjected to Rietveld analysis. In due course, seven literature points were used to set up a plot of C/Zr ratio *vs.* the cell parameter (*cf.* Fig. 3) and the data polynomially fitted. The cell parameters from our samples were then subjected to the same fitting routine and thus, the C/Zr ratio re-calculated. These ratios are then used for labelling the different zirconium oxycarbide materials according to their C/Zr ratio.

The XRD data depict all signals for a phase-pure oxycarbide similar to the oxycarbide reference with the composition “ZrC_0.71_O_0.29_”.^24^ Some amorphous broad humps are related to silicone grease, which was used between two acetate foils to prepare the powder samples for the XRD measurement. The sample with a C/Zr ratio of 0.63 (black colored line) shows signals of residual cubic, monoclinic and tetragonal ZrO_2_ phases. This higher oxidized material shows signals for the oxycarbide phase shifted to higher 2*θ* values. The overlapping peaks of this signal gradually separate to higher 2*θ* regions into a peak and a shoulder. This indicates a mix of similar oxycarbide species with different lattice parameters, which results from a different carbon/oxygen composition. Crystallographic data show a smaller unit cell parameter for higher oxidized species. This implies that some crystallites with a higher oxygen content and the same *Fm*3[combining macron]*m* unit cell exist in the powder.

As introduced above, Rietveld refinement for the XRD data of the phase-pure zirconium oxycarbides with different C/Zr ratios has been performed with a Thompson-Cox-Hastings profile function, which is optimized for the used XRD device (the one with a C/Zr ratio of 0.71 is exemplarily shown in Fig. 2). Additionally, a calibration for the correlation of the C/Zr ratio with the unit cell parameter was created with all available ICSD oxycarbide data.^9^,^23^,^24^ The carbon content is then plotted *vs.* the cell parameter and polynomially fitted (Fig. 3). The calculated cell parameters of the four synthesized oxycarbide powders, which were obtained by Rietveld refinement, are appended to the fit curve to re-calculate the C/Zr ratio. The calculated cell parameters and composition (with a standard deviation *s* = ± 0.05) for the powder sample are summarized in Table 1.

**Table 1:** Rietveld data (calculated cell parameters and C/Zr ratio) for the four synthesized Zr oxycarbide materials

C/Zr ratio	Cell parameter	Crystal density
0.63	4.6565 Å	6.76 g cm^–1^
0.66	4.6639 Å	6.74 g cm^–1^
0.69	4.6689 Å	6.73 g cm^–1^
0.73	4.6753 Å	6.71 g cm^–1^

**Fig. 4:**
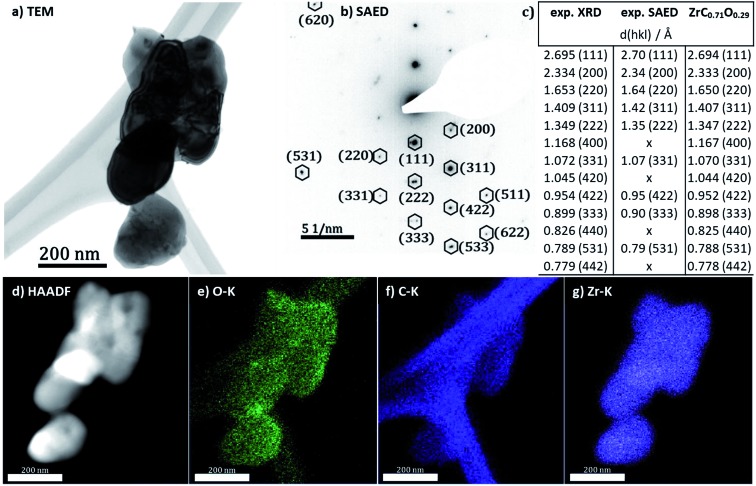
(a) TEM overview of agglomerated zirconium oxycarbide particles, synthesized by the hybrid gelation method. (b) Corresponding selected area electron diffraction (SAED) pattern of that area. All reflexes can be attributed to a structure with a C/Zr ratio of 0.71. (c) Summary of the most significant *d*-space values associated and ordered by lattice planes. X-ray diffraction and electron diffraction are compared to the reference structure. (d) HAADF image. (e–g) Elemental EDX maps (O–K, C–K and Zr–K edges) of the zirconium (oxy)carbide particles. The carbon region shows, besides the signal from the lacey carbon film, also clearly the oxy-carbide related signal.

For a better visualization of the oxycarbide powder and a further structural and elemental analysis, transmission electron microscopy and spectroscopy results are displayed in Fig. 4. For the sake of comparability, all analyses were taken from the same spot at the powder. The bright field TEM image in Fig. 1a shows a cluster of agglomerated zirconium oxycarbide powder particles. The size of the particles is in the range of a few 100 nm in diameter, while the morphology generally appears roundish. The particle interface region reveals some fringes. These quite common contrast oscillations can be explained by the thickness effect,^25^ meaning that the diffraction interference changes with a not uniform thickness, in this case the declining thickness near the edge of the particles.

Structural and phase analysis of the zirconium oxycarbide powder by SAED (Fig. 4b) reveals that in fact all reflections could be unequivocally assigned to the same zirconium (oxy)carbide structure. In fact, the combined analysis of X-ray diffraction and electron diffraction (Fig. 4c) is in good accordance. To further reveal details about the chemical homogeneity of Zr, C and O, HAADF imaging (Fig. 4d) is combined with elemental analysis by EDX (Fig. 4e–g). All elements are homogeneously distributed within the sample bulk, which is especially true for oxygen and carbon. Note that the distribution of carbon is slightly hampered by the strong signal of the lacey carbon film used to support the powder in the microscope. A definite qualitative judgement of the carbon signal from the sample powder, is however possible. According to BET and BJH measurements, the oxycarbide powder (including carbon excess) has no pores and a specific surface of 350 m^2^ g^–1^.

**Fig. 5:**
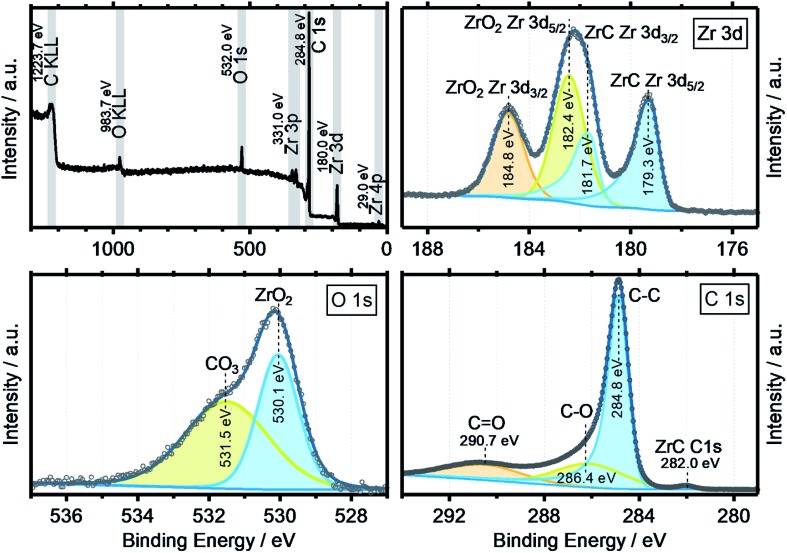
XPS survey spectrum (top left) and detailed spectra for Zr 3d (top right), O 1s (bottom left) and C 1s (bottom right) from the powder surface of the synthesized zirconium oxycarbide with the C/Zr ratio of 0.69.

XPS data were obtained to get a more complete image of the oxycarbide chemistry. Fig. 5 shows the collective XPS results as detailed spectra for the Zr 3d, O 1s and C 1s binding energy regions. The survey spectra show all signals for the elements zirconium, oxygen and carbon, including the two Auger peaks O KLL and C KLL with other impurity elements well below the detection limit of the spectrometer. The detailed XP spectra of the zirconium 3d region shows the typical spin–orbit splitting for both the ZrC (179.3 eV) and ZrO_2_ (182.4 eV) components by 2.4 eV, with the correct area ratio of 3 : 2 for the 3d_5/2_ to 3d_3/2_ orbitals.^26^ No suboxides or metallic zirconium features were found, which indicates a fully oxidized surface with no, or little lattice defects. In the O 1s spectra, the two components ZrO_2_ (530.1 eV) and a surface-bound Zr carbonate species (531.5 eV) were deconvoluted from the experimental data. The surface chemistry seems to be dominated by fully oxidized zirconium and oxidized carbon, especially carbonate species. It is expected that the ZrO_2_ at the surface results from a reoxidation process of the oxycarbide, which has been previously also reported for other titanium oxycarbides.^27^ In case of titanium (oxy)carbide with a C/Ti ratio of 0.5, the re-oxidation has been shown to be caused by the thermodynamic instability of the oxycarbide phase and reaction to TiO_2_ and carbon at the very surface, through contact with air and anodic polarization in an acidic electrolyte.

The C 1s region features, besides residual carbon and different adventitious oxidized carbon species as a result of air transport, also a zirconium carbide (ZrC) peak at 280.0 eV.^26^

## 4. Conclusion

We have demonstrated the unique possibilities of a hybrid solid–liquid synthesis routine to zirconium-based (oxy)carbide materials, a class of solids with emerging technological perspectives. Usually only formed under rather harsh experimental conditions, our approach reproducibly delivers phase-pure zirconium (oxy)carbide nanopowders at surprisingly low preparation temperatures. Combined structural and spectroscopic information confirms the presence of an (oxy) carbide material. The proposed synthesis routine opens new possibilities in the controlled preparation of oxycarbide materials with defined C/Zr ratio, as well as an in-depth physico-chemical characterization of their intrinsic properties, such as the conductivity. Especially the latter for similar oxycarbide materials has been shown to be crucial for technological use, *e.g.* as anode material in solid-oxide fuel cells, where a high ionic conductivity is desirable.

## Conflicts of interest

There are no conflicts to declare.

## References

[cit1] Greenwood N. N. and Earnshaw A., Chemistry of the elements, Pergamon Press, Oxford, 1st edn, 1994.

[cit2] Wan W., Tackett B. M., Chen J. G. (2017). Chem. Soc. Rev..

[cit3] Réjasse F., Rapaud O., Trolliard G., Masson O., Maître A. (2016). RSC Adv..

[cit4] Hou X., Marin-Flores O., Kwon B. W., Kim J., Norton M. G., Ha S. (2014). J. Power Sources.

[cit5] Sinha A., Miller D. N., Irvine J. T. S. (2016). J. Mater. Chem. A.

[cit6] Pierson H. O., Handbook of refractory carbides and nitrides. Properties, characteristics, processing and applications, Noyes publication, Westwood, N.J., 1996.

[cit7] Shul’pekov A. M., Lyamina G. V. (2011). Inorg. Mater..

[cit8] Modine F. A., Foegelle M. D., Finch C. B., Allison C. Y. (1989). Phys. Rev. B: Condens. Matter Mater. Phys..

[cit9] Gendre M., Maître A., Trolliard G. (2011). J. Eur. Ceram. Soc..

[cit10] Yan Y., Huang Z., Liu X., Jiang D. (2007). J. Sol-Gel Sci. Technol..

[cit11] Maitre A., Lefort P. (1997). Solid State Ionics.

[cit12] Berger L.-M., Gruner W., Langholf E., Stolle S. (1999). Int. J. Refract. Met. Hard Mater..

[cit13] Abdelkader A. M. (2016). J. Eur. Ceram. Soc..

[cit14] Abdelkader A. M., Fray D. J. (2012). J. Eur. Ceram. Soc..

[cit15] Liu H., Cai Y., Xu Q., Liu H., Song Q., Qi Y. (2017). RSC Adv..

[cit16] Pang Z., Zou X., Zheng K., Li S., Wang S., Hsu H.-Y., Xu Q., Lu X. (2018). ACS Sustainable Chem. Eng..

[cit17] Danks A. E., Hall S. R., Schnepp Z. (2016). Mater. Horiz..

[cit18] Yan C., Liu R., Cao Y., Zhang C., Zhang D. (2012). J. Sol-Gel Sci. Technol..

[cit19] Shannon R. D. (1976). Acta Crystallogr., Sect. A: Cryst. Phys., Diffr., Theor. Gen. Crystallogr..

[cit20] Pan L., Heddy R., Li J., Zheng C., Huang X.-Y., Tang X., Kilpatrick L. (2008). Inorg. Chem..

[cit21] Weiss I. M., Muth C., Drumm R., Kirchner H. O. K. (2018). BMC Biophys..

[cit22] Desimoni E., Casella G. I., Cataldi T. R. I., Malitesta C. (1989). J. Electron Spectrosc. Relat. Phenom..

[cit23] Barnier P., Thévenot F. (1986). Int. J. High Technol. Ceram..

[cit24] Constant K., Kieffer R., Ettmayer P. (1975). Monatsh. Chem..

[cit25] Williams D. B. and Carter C. B., Transmission Electron Microscopy. A Textbook for Materials Science, Springer US, Boston, MA, 2nd edn, 2009.

[cit26] Moulder J. F. and Chastain J., Handbook of x-ray photoelectron spectroscopy. A reference book of standard spectra for identification and interpretation of XPS data, Physical Electronics Division, Perkin-Elmer Corp, Eden Prairie, Minn., 1992.

[cit27] Calvillo L., Fittipaldi D., Kunze-Liebhäuser J., Pacchioni G., Granozzi G. (2014). J. Phys. Chem. C.

